# Increasing persistency in lay and stabilising egg quality in longer laying cycles. What are the challenges?

**DOI:** 10.1080/00071668.2016.1161727

**Published:** 2016-05-23

**Authors:** M. M. Bain, Y. Nys, I.C. Dunn

**Affiliations:** ^a^ IBAHCM, College of MVLS, University of Glasgow, Bearsden, Glasgow, Scotland, UK; ^b^ INRA, UR83 Recherches Avicoles, Nouzilly, France; ^c^ Roslin Institute and Royal (Dick) School of Veterinary Studies, University of Edinburgh, Easter Bush, Midlothian, Scotland, UK

## Abstract

1. In the past 50 years, selection starting initially at the breed level and then using quantitative genetics coupled with a sophisticated breeding pyramid, has resulted in a very productive hybrid for a variety of traits associated with egg production.
2. One major trait currently being developed further is persistency of lay and the concept of the “long life” layer. Persistency in lay however cannot be achieved without due consideration of how to sustain egg quality and the health and welfare of the birds in longer laying cycles. These multiple goals require knowledge and consideration of the bird’s physiology, nutritional requirements, which vary depending on age and management system, reproductive status and choice of the selection criteria applied.
3. The recent advent of molecular genetics offers considerable hope that these multiple elements can be balanced for the good of all in the industry including the hens.
4. The “long life” layer, which will be capable of producing 500 eggs in a laying cycle of 100 weeks, is therefore on the horizon, bringing with it the benefits of a more efficient utilisation of diminishing resources, including land, water, raw materials for feed as well as a reduction in waste, and an overall reduced carbon footprint.

## INTRODUCTION

**Figure 1.**
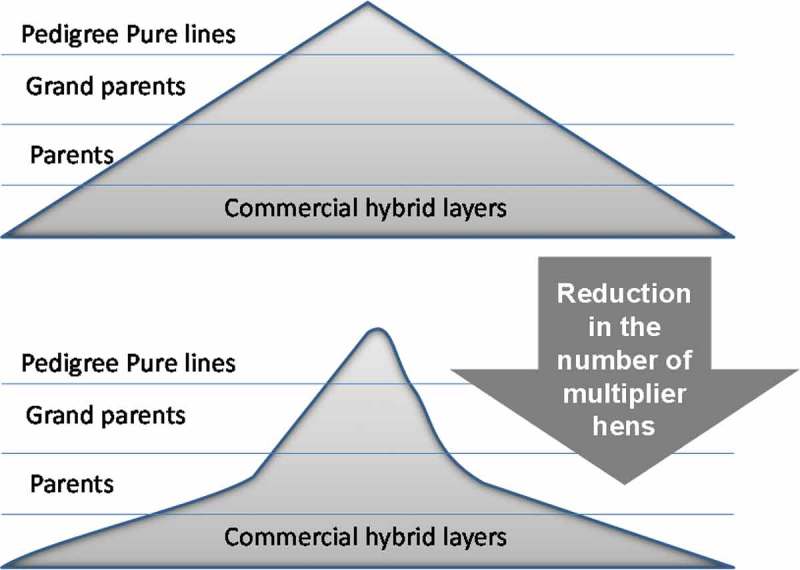
Stylised representation of how increasing the laying cycle to 90–100 weeks will significantly reduce the number of multiplier hens and hence the overall shape of the breeding pyramid.

Over the next 4 decades, the world’s population is forecast to increase by 25% and to meet this challenge food production needs to increase by 60% (FAOSTAT, 2013). Eggs constitute one of the most affordable sources of animal protein available, and so it is not surprising that the number of laying flocks is rapidly increasing in developing countries like India and China. In Europe, the priority is to increase egg production by breeding for increased persistency in lay and stability in egg quality so that the laying cycle of commercial flocks can be extended to 90–100 weeks. Although making hens lay for longer may not receive universal acclaim from those opposed to current farming practices, it appears to be the most logical approach to the efficient utilisation of resources as the benefits are both financial and environmental. For example, we estimate that even 25 more eggs per hen could potentially reduce the UK flock, including breeding hens, by 2.5 million birds per annum. The net effect of this on the overall shape of the breeding pyramid is illustrated in Figure 1. The potential savings highlight the environmental benefits of a reduction in the national breeding flock as well as the obvious reduction in food required to maintain these hens. On the environmental front it was calculated that around 1 g of nitrogen could be saved per dozen eggs for an increase of 10 weeks in production (Dr Murdo Macleod, personal communication). This would considerably reduce the nitrification impact of increasing or maintaining production that is especially important in nitrate sensitive areas.

Breeding companies claim that they will have developed the “long life” layer which will be capable of producing 500 eggs in a production cycle lasting 100 weeks by 2020 (Van Sambeek, 2010; Hyline International, 2014). This goal is being achieved using selection programmes that base decisions on a triangulation of phenotype evaluation of pure lines extending beyond 55 weeks, cross breed progeny testing (which is now being carried out in diverse conditions throughout the world) and genotype information derived from DNA markers (microsatellite and SNP), which have been validated to show an association with phenotypic traits (O’Sullivan, 2009). Any improvement in persistency in lay however must also go hand in hand with sustainable egg quality and the birds must remain healthy throughout the production period. A decline in egg numbers combined with a deterioration in shell quality are the main reasons for currently replacing flocks at or around 72 weeks of age. Poor shell quality at 72 weeks does not mean that all hens in an ageing flock produce eggs of reduced quality, rather the variability in egg quality within the flock increases. The long-term maintenance of the tissues and organs involved in producing eggs is therefore a prerequisite for extending the laying cycle of commercial flocks (Dunn, 2013). However, despite a plethora of research in this area spanning over 50 years, we are still ignorant of all the processes and mechanisms controlling the complexity of egg formation, nor do we fully understand the functional properties of the individual components of the egg, which are proving to be much more intricate than we ever imagined. Three excellent reviews on these subjects are provided by Nys and Guyot (2011), Rehault-Godbert *et al*. (2011), and Hincke *et al*. (2012).

Osteoporosis remains one of the major welfare challenges for the egg industry (Sandilands, 2011) and therefore cannot be ignored in any discussion relating to extending the laying cycle. In this respect, the correct nutrition throughout the laying cycle is of paramount importance. The nutritional requirements of the “long life” layer therefore also needs to be critically evaluated as the results of nutritional trials carried out over 20 years ago (when birds produced fewer eggs) may no longer be directly applicable.

This review paper begins with an overview of egg formation and some of the main factors that control or influence this process. A summary of the progress made by breeding companies in achieving their aim of improving persistency in lay and stabilising egg quality is then provided. The final part of the paper looks at some of the nutritional and welfare considerations which need to be addressed if the concept of extending the laying cycle beyond 72 weeks is to be realised in practice.

## OVERVIEW OF THE EGG FORMING PROCESS

The almost daily production of an egg by a commercial layer is only feasible due to the simultaneous development of a series of follicles in the left ovary. This follows a defined hierarchy with only one follicle reaching maturation within each 24 h period. Over 12000 oocytes are present in the ovary at hatch but only a small percentage of these will ever reach maturity. At ovulation, the yolk mass from the largest follicle is captured by the funnel shaped open end of the proximal oviduct, the infundibulum. From here, it travels down the oviduct and undergoes successive deposition of the different components of the egg (Romanoff and Romanoff, 1949; Gilbert, 1979; Sauveur and De Reviers, 1988). Each component of the egg (the albumen, membranes and the shell) is secreted by different parts of the oviduct according to a predetermined sequence of events. During the first 4 h, the egg white (albumen) is formed in the magnum, the longest and most glandular region of the oviduct. The shell membranes are then deposited as the forming egg mass passes through the isthmus. Five h after ovulation, the egg enters the shell gland, where it spends the next 19 h. It is during this time that the shell forms. The formation of the eggshell occurs in 3 distinct phases (Nys *et al*., 2004) and is regulated by the precise temporal and spatial secretion of a complex array of organic matrix proteins (Gautron *et al*., 1997; Mann *et al*., 2006), some of which subsequently become incorporated into the calcified structure thereby modifying its biomechanical properties (Hincke *et al*., 2012) and/or participate in its antimicrobial defences (Rehault-Godbert *et al*., 2011). The resulting interwoven fabric of organic and inorganic constituents forms the mammillary and palisade layers of the shell. In the last 1.5 h and just prior to oviposition, the shell pigment and finally the cuticle (a non-calcified organic layer of variable thickness) are deposited. The egg is then ready for oviposition. The timing and process of oviposition is controlled by neurohypophyseal hormones and prostaglandins secreted by the ovary and to a lesser extent the shell gland (Nys and Guyot, 2011). Hens normally ovulate and oviposit one to 7 h after dawn when on a standard 14L:10D lighting programme; therefore, hens lay their egg in the first hours of the light period. The next ovulation takes place after expulsion of the egg but can also occur just prior to this in some cases (Nys and Guyot, 2011).

## THE ROLE OF THE NEUROENDOCRINE SYSTEM

**Figure 2.**
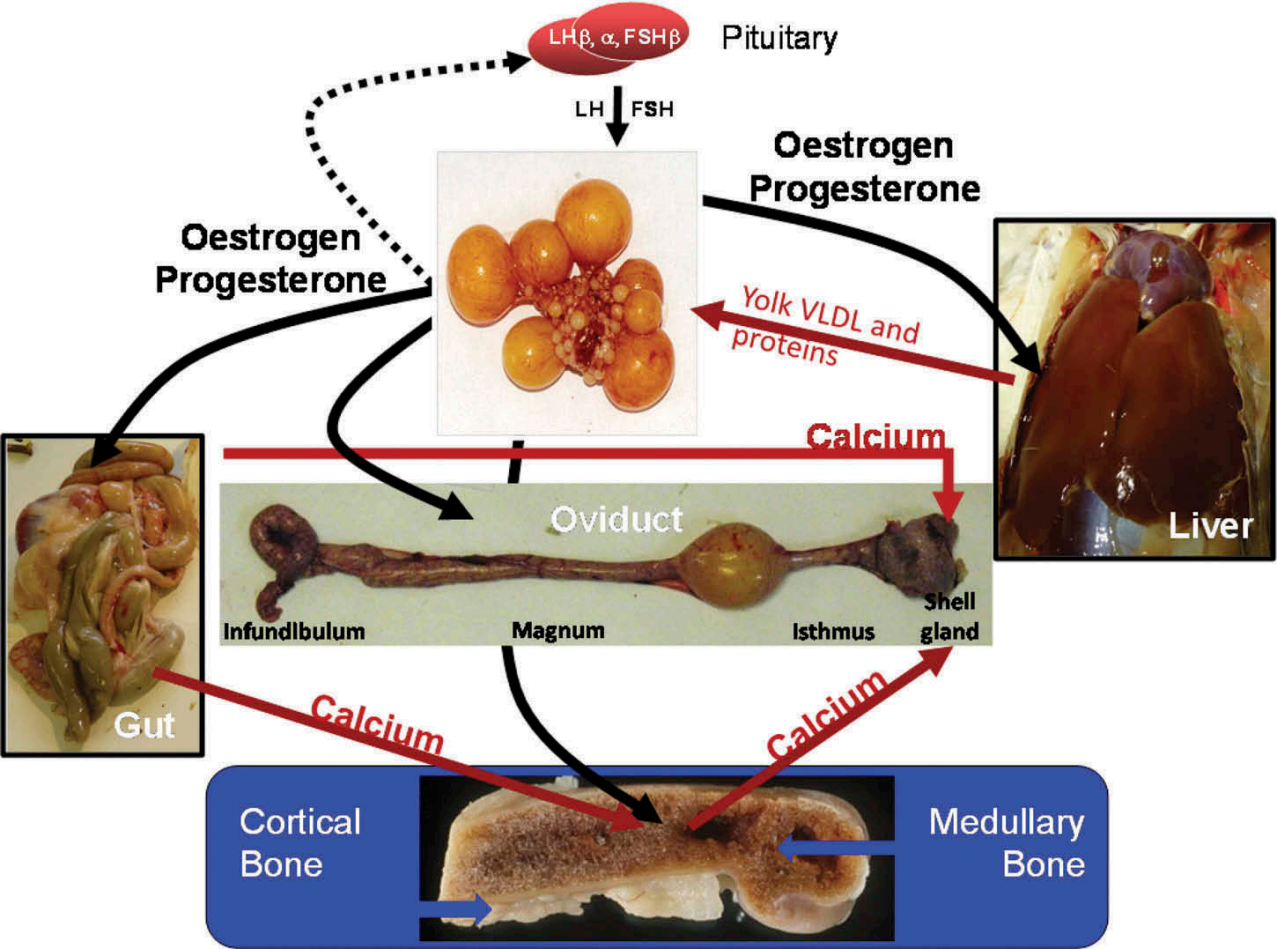
Simplified summary of the endocrine control of the principle components of egg formation. Reproduction is ultimately controlled by GnRH-I neurones in the hypothalamus region of the brain which integrate environmental and internal endocrine signals (not shown). GnRH-I peptide released from the median eminence of the hypothalamus stimulates the pituitary gland (represented at the top of the diagram) to release luteinising hormone (LH) and follicle stimulating hormone (FSH). These gonadotrophins stimulate the development and growth of follicles in the left ovary. The developing follicles in turn secrete the sex steroids, oestrogen and progesterone, which are responsible for the spectacular growth of the oviduct which produces the egg white, membranes and shell. Oestrogen and progesterone also have a direct effect on the liver by initiating synthesis of the various yolk constituents and on the gut by enhancing the uptake of dietary derived calcium. Furthermore osteoblasts in the long bones start forming medullary bone rather than structural bone in response to oestrogen. The rate at which calcium is removed from the blood during egg shell formation is greater than the mean rate of calcium absorption from the diet, and the balance is made good by the mobilisation of principally medullary bone reserves.

Reproduction in birds is controlled by GnRH-I neurones in the hypothalamus, the region of the brain that integrates environmental and internal endocrine signals. Dunn (2013) suggested that subtle differences in the neuroendocrine system between individuals may be the reason why some birds are capable of a higher persistency of lay than others. Oestrogen and progesterone are critical to stimulating the growth and maintenance of the left oviduct (Sharp *et al*., 1992). These sex steroids are produced by the developing follicles in the ovary at sexual maturity in response to an increase in the circulating levels of gonadotrophins such as pituitary luteinising hormone (LH) and follicle stimulating hormone (FSH) as indicated in Figure 2. Oestrogen also plays an important role in the formation and maintenance of medullary bone in the marrow cavity of long bones at the onset of lay (Dacke *et al*., 1993) As hens age, the cells in the hypothalamus that control these processes are thought to become less efficient (Dunn *et al*., 2009). The net effect is that the oviduct loses weight, and functions less efficiently. The oviduct itself must inevitably suffer damage due to wear and tear, possible low grade infections, and probably becomes refractory to the prolonged stimulation (Dunn, 2013). The number of days when no egg is laid subsequently increases as does the number of defective eggs (Solomon, 1991, 2002). However, some individuals are clearly more capable of maintaining a high egg output with good quality shells for longer periods. Thus, improving persistency in lay and sustaining egg quality in longer laying cycles should be achievable.

## SELECTION AND MAINTAINING LAYING PERSISTENCY

The intensive selection for traits such as age at sexual maturity, peak production and laying persistency to 55 weeks has significantly reduced the genetic and phenotypic variations that previously existed in egg number in commercial lines. Indeed, the biological limit of one egg per day for example has virtually been achieved at peak production. It is now common practice for breeding companies to extend their pure line evaluation beyond 75 weeks. Heritability calculations for egg production at 80–100 weeks are reported to be moderate (*h* ^2^ = 0.24) for both white and brown egg layers (Institut de Sélection Animale, personal communication). There is therefore further scope for genetic improvement in laying persistency.

## SELECTION TO STABILISE EGG QUALITY IN LONGER-LAYING CYCLES

For many years, breeding companies have focused their efforts on achieving higher egg weights (60 g) by peak production and maintaining egg weight at or around this level for as long as possible (65.5 g by 50 weeks). Beyond this, egg weight creeps up with bird age whilst shell quality tends to deteriorate. Excessively large eggs must be avoided if the laying cycle is increased, as large eggs are notoriously difficult to handle. The selection has initially focused on controlling egg weight after peak production and keeping egg weight stable beyond 90 weeks of age. The net effect is that the shape of the egg weight curve has become flatter, and “late egg size” has decreased by 5–7 g (O’Sullivan, 2009).

The Haugh unit is the standard selection measurement for albumen quality. Curtis *et al*. (2005) reported that Haugh units deteriorate with hen age from an average 89.6 to 68.8 over the laying period. The heritability estimates for Haugh units range from 0.21–0.41 (Dunn, 2011). The heritability estimates for Haugh units calculated over a longer laying cycle at 80–100 weeks are still within this range (Institut de Sélection Animale, personal communication). Thus, through selection it is also possible to maintain acceptable albumen quality in older laying flocks for a longer period in the future.

Egg colour is only included in selection in brown egg laying populations for aesthetic reasons and not because this trait relates to the quality of the egg in any other way. The natural variation in brownness is considered to be important in some markets but this is not universal (Arthur and O’Sullivan, 2005). Heritability for shell colour in brown lines ranges from 0.3–0.53 depending on the breed (Dunn, 2011).

Eggshell strength on the other hand is vital in ensuring the integrity and safety of the egg contents but the problem here is deciding on which measurement to use. Most companies use a combination of several measurements as they believe that each measures slightly different things. One breeding company for example uses puncture strength as a measure of flexibility and breaking strength as an indirect measure of shell thickness. Heritability estimates for breaking strength measured by quasi-static compression in brown and white lines have been reported to be 0.28 at 80–100 weeks (Institut de Sélection Animale, personal communication). Thus, sustained eggshell strength in older flocks is also a realistic and achievable goal.

Some breeding companies have recently introduced the measurement of dynamic stiffness into their breeding programmes. This measurement was developed by researchers at KU Leuven in the late 1990s. The dynamic stiffness measurement was shown by Dunn *et al*. (2005) to have a high heritability and importantly the measurement was shown to be an accurate predictor of an egg’s susceptibility to damage in the field (Bain *et al*., 2006). This unique feature means that breeding companies (who have been using this measurement for several years now) are reporting beneficial effects at the commercial level in terms of a measurable reduction in the percentage cracks and also in the breeding sector where improved eggshell quality has resulted in improved hatchability (Lohmann Tierzucht, personal communication).

## NUTRITION AND FEEDING FOR PERSISTENCY IN LAY AND GOOD EGG QUALITY

It is important that pullets receive an appropriate diet throughout the rearing phase so that they meet the recommended adult pullet target weight by 14–16 weeks of age and have the correct body composition to sustain egg production beyond 90 weeks. A specific growth curve must therefore be followed. This is particularly important in the case of the “long life” layer where persistency in lay is expected. Any deviation away from the target pullet weight will influence the mean egg weight during the early laying phase (*r* *^2^* = 0.85, *P < *0.01) and the total egg output for the entire period of production (Bouvarel *et al*., 2011). Particular attention must be paid to the energy/ protein ratio between 11–16 weeks, as increased energy content of the diet enhances the fattening score (Cheng *et al*., 1991). Particle size, if not suitable for beak size, can also result in reduced feed intake and therefore weight gain during the rearing phase (Frikha *et al*., 2011). Too many dietary changes or rapid changes in diet during the rearing phase should also be avoided. At about 16 weeks of age, the energy and protein content of the ration must be adjusted to ensure that the hen consumes sufficient feed to cope with growth and the onset of egg production. There are a number of ways of promoting feed intake around this time, for example use of whole cereals and coarse water-insoluble fibre (Hetland *et al*., 2005). It is particularly critical that this feed is both appetising and always available as medullary bone reserves are being formed and the ovary and oviduct are developing at this time.

During the laying period, the first challenge is to adjust the energy and protein requirements to optimise egg output and to carefully control body weight. The growth requirement is only present for the first few weeks at the onset of egg production. Energy required for maintenance thereafter depends on body weight and feather coverage and therefore increases with hen age. Compilation of literature clearly shows a strong and negative correlation between feed intake and dietary energy concentration (Bouvarel *et al*., 2011). This adaptation is however only partial and so high energy diets can be used during the first part of the laying period to satisfy the continued requirement for growth and to promote heavier, early egg weight without the risk of overfeeding and producing “fat hens” (Perez-Bonilla *et al*., 2012). The hen’s energy requirement however decreases as egg production becomes established. To minimise fat deposition a lower energy diet can be used at this time, as the birds will be able to partially compensate by increasing their feed intake. Laying hens also adjust their food intake according to the relative size of the particles in relation to the beak size (Joly, 2004; Safaa *et al*., 2009). Varying particle size allows further balancing of the energy intake.

The crude protein concentration and amino acids in the layer diet are also important, methionine being the main limiting amino acid (AA). Consumption of an extra 1 g of protein per day for example results in an average increase in egg weight of 1.4 g (Bouvarel *et al*., 2011). However, the amount of protein consumed is dependent on the dietary energy concentration and the form of the ration. Ideally, the protein and AAs concentration in the diet should be estimated relative to the egg weight (mg/g of egg for AAs) and adjusted to optimise egg production throughout the laying cycle. However, an additional difficulty is that the heterogeneity of the flock increases with flock age. The best strategy is therefore to focus on maintaining the production of the higher producing hens and to adjust the supply of proteins and AAs accordingly, providing the cost is not prohibitive.

“Gap” feeding where there is a 3 to 4 h gap between feedings allowing birds to consume fine particles, is a useful management tool to improve feed intake efficiency, and flock body weight uniformity during the laying period (Hyline International, 2015). Sequential feeding methods whereby the energy and protein levels in the morning versus the afternoon feed are varied are also under investigation (Traineau *et al*., 2013, 2015). Knowledge of the hen’s specific needs for energy and protein throughout the day would allow optimisation of the daily intake and improved FCR. At present, however, there seems to be no clear evidence that the hen’s requirement for energy or protein varies throughout the day and that laying hens can adjust their daily intake accordingly. In contrast, the laying hen’s specific appetite for calcium in the late afternoon is well established (Mongin and Sauveur, 1979).

## DIETARY CALCIUM, EGGSHELL QUALITY AND BONE HEALTH IN LONGER-LAYING CYCLES

A laying hen requires 2.2 g of calcium on average for every egg she lays. About two-thirds of this calcium can be directly supplied via the hen’s diet, and one third by the mobilisation of calcium from the medullary bone that forms under the influence of oestrogen as the bird first comes into lay (Bouvarel *et al*., 2011). Calcium derived from bone is needed during the final stages of shell formation as this takes place during the night when the bird is not feeding. Medullary bone, unlike structural bone, is capable of undergoing rapid absorption and renewal. Unfortunately, resorption of structural bone also occurs causing the symptoms of osteoporosis (Whitehead, 2004). Osteoporosis is caused by a decrease in the amount of fully mineralised structural bone leading to bone fragility and susceptibility to fracture making this one of the major welfare challenges for the egg production industry (Sandilands, 2011). Nevertheless, within a flock it is possible to observe individual birds with high productivity and good bone strength (Dunn *et al*., 2007). Genetics has been shown to be important, with around 40% of the variation in an index of bone quality being due to genetics (Bishop *et al*., 2000). This bone quality index was used successfully to improve bone strength and reduce skeletal damage (Fleming *et al*., 2004). Unfortunately, the measurement of the trait requires the hen to be killed and is not practical under commercial conditions, but advances in selection technologies may soon address this. Therefore, it should be possible to select for improved bone strength in the “long life” layer but since the aetiology of osteoporosis is complex and is not only influenced by genetics but also by the laying environment and by nutrition, genetic selection alone is not the answer (Fleming *et al*., 2006).

The provision of insufficient dietary calcium during the rearing or laying period has an adverse effect on both eggshell quality (Classen and Scott, 1982; Hartel, 1990) and bone strength (Whitehead, 2004). The laying hens requirement for dietary calcium within the diet for different ages is in the order of 0.9 to 1.2% during the growth period of the pullet, increasing to 2 to 2.5% just prior to the onset of lay and 3.5 to 4.5% once lay is established (Bouvarel *et al*., 2011). There does not appear to be any benefit of adding more calcium or using a “step up” feeding system (3.5, then 4.5, and finally 5.5%) to limit the age deterioration in shell quality (Keshavarz and Nakajima, 1993). Improving bone reserves of calcium relies more on the form of calcium supplied and on the use of large particles of calcium (Guinotte and Nys, 1991).

Most eggs are laid early in the morning just after the lights come on. Consequently, the morning feed probably does not contribute directly to shell formation as this does not commence for 5 h after oviposition. Likewise, the afternoon feed is not synchronised with shell formation as this continues throughout the night; however, a specific appetite for calcium a few hours before the lights go off does ensure that there is some storage of feed including calcium in the crop (Mongin and Sauveur, 1979). Switching on the lights for 2 h and introducing a midnight feed has been shown to improve the synchronisation of dietary calcium intake with shell formation and to improve eggshell quality (Grizzle *et al*., 1992). However, the European directive 1999/74/CE requires birds to have 8 h of uninterrupted darkness in every 24 h period, and so this type of split lighting programme is essentially prohibited in Europe. Studies by Keshavarz (1998) showed that the daily calcium requirement cannot be reduced by providing hens with adequate levels of calcium during the afternoon and inadequate calcium level during the morning. Calcium provided in the morning feed is therefore probably important for replenishing medullary bone calcium reserves.

In order to optimise eggshell quality, the calcium particle size should be adjusted according to the density and solubility of the particulate calcium source (quarry or marine). As a general rule a coarse particle of 1–2.4 mm with a low solubility should be introduced to supply two-thirds of the calcium along with a highly soluble marine source as particles of 2–4 mm (Bouvarel and Nys, 2013). Dietary lipid content and the active metabolite of vitamin D3 have been shown to influence the efficiency of dietary calcium uptake in the gastrointestinal tract but how this relates to the transfer of calcium in the shell gland remains to be determined. High levels of phosphorus or too much or too little salt (NaCl) in the layer diet also has a deleterious effect on eggshell quality and should be avoided (Bouvarel and Nys, 2013). In summary, there is still much to learn about the kinetics of intestinal calcium retention throughout the day and such knowledge will clearly be of value in the management of “long-life” layers.

## NUTRITIONAL CHALLENGES – PREVENTION OF METABOLIC DISEASE

The export of large amounts of protein and lipid into eggs during the laying period challenges hen metabolism and can also cause a range of other metabolic diseases. In birds, all fatty acid synthesis occurs in the liver, mostly from carbohydrate. The egg yolk contains a large amount of lipid. The metabolic activity of the liver therefore has to dramatically increase from sexual maturity onwards to form the lipoprotein precursors of the yolk (Nys and Guyot, 2011). Fatty acid synthesis is coupled with glycolysis so a high dietary consumption of carbohydrate can markedly increase the fat content of the liver resulting in hepatic steatosis (Butler, 1976; Hansen and Waltzem, 1993). Hepatic steatosis in layers tends to occur most often where there is an imbalance in the protein/ energy ratio and causes a drop in egg production and obese hens. Fatty liver haemorrhagic syndrome (FLHS) has also been observed in hens treated with oestrogens (Butler, 1976; Lee *et al*., 2010; Choi *et al*., 2012). This disease, which is not to be confused with hepatic steatosis, is almost invariably fatal but it can also affect egg production (Walzem *et al*., 1993; Lee *et al*., 2010). The provision of dietary 25 (OH) D3, feed restriction with substitution of carbohydrate by dietary fat, supplementation of choline, inositol, vitamin B12, folic acid and vitamin E in the diet have all been shown to limit the incidence of hepatic steatosis and FLHS in layers (Bouvarel and Nys, 2013).

## DISEASE, ENVIRONMENTAL STRESS AND BIRD BEHAVIOUR

Both disease and environmental stress can induce changes in egg formation at any time during the laying year (Solomon, 1991). Diseases, such as infectious bronchitis, egg drop syndrome and Newcastle disease influence egg quality either directly by altering oviduct structure or indirectly by lowering the general health status of the individual. Remedial action in such cases often involves medicating or vaccinating the entire flock with variable success. Within the laying environment, individual birds also experience a range of “stress” events of varying magnitude and duration within any 24 h period. These stress events are likely to be exacerbated in large size, colony management systems where birds are not able to establish a stable dominance hierarchy. When these “stress” events coincide with a critical point in the egg forming process the result will be the production of an egg of unacceptable quality. Experimentally, it has been shown that the oviduct can recover even after experiencing a major stress event, and catastrophic damage to the cells lining the oviduct (Watt, 1989; Solomon, 2002). The recovery process however can take 2–3 weeks. There is no “magic cure” for environmental stress and in many cases, it is impossible to identify the stressor and therefore redress the balance especially at the individual bird level.

Feather pecking in layers is a very clear welfare problem in non-cage housing systems with a range of prevalences of 40%–80% (Blokhuis *et al*., 2007). Feather pecking results in feather loss and in extreme cases causes damage to the bird receiving the behaviour and ultimately their death due to cannibalism. Poor feather coverage also means that birds need to eat more to maintain their body temperature (+20 g at 12.8°C if the feather coverage corresponded to 50% normal, +7 g at temperature of 23.9°C) (Peguri and Coon, 1993). Feather pecking is therefore an important welfare and economic issue (Blokhuis *et al*., 2007) and one that the industry needs to address, particularly if there is an industry wide ban on beak trimming (the main method used to control this behaviour). Indeed, following an EU welfare directive on the issue, beak trimming has already been banned in some European countries and others are working towards this. It is therefore important to be able to identify practical, effective and affordable alternatives to beak trimming. The factors involved in feather pecking outbreaks in commercial flocks are multifactorial, for example nutrition seems to be important. Reports suggest that feather pecking behaviour can be reduced by feeding diets high in insoluble non-starch polysaccharides (Van Krimpen *et al*., 2007, 2009) or roughage (Steenfeldt *et al*., 2007) and by avoiding protein or amino acid deficiency. Feather pecking behaviour has also been observed in pullets as young as 7 d old. Limiting this behaviour through nutritional means during the early stages of pullet rearing might therefore be important in trying to reduce feather pecking in adult birds (Qaisrani *et al*., 2013). Feather pecking also has a genetic basis as demonstrated by Kjaer *et al*. (2001) and others but with variable heritabilities. There is also an interesting facet in that individuals can affect each other’s phenotype – this indirect genetic effect has led to promising research on selection by family group (Peeters *et al*., 2012). The first genetic regions (QTL) involved in feather pecking have now been reported (Rodenburg et al., 2004) thus molecular genetics once again offers poultry breeders potential solutions to this intractable problem.

## CONCLUSIONS

The economic forces for continuing improvement in productivity are large but there is also a demand for improved welfare.

The benefits of genetic selection for improved persistency in lay and stability in egg quality whilst maintaining skeletal health can only be realised if they are matched by improvements in hen nutrition and careful monitoring, recording and analysis of the effects of this process on the health and welfare of the hens. The opportunities for broader selection indices, linked to molecular genetics, are increasing and it is evident that poultry geneticists are already harnessing this added tool in an attempt to address some of the major issues that the industry faces for the betterment of the hens, the consumer and the environment. To reach the goal of a long life layer a multifactorial approach including genetics, nutrition and design of housing systems will need to be adopted to ensure that effects on correlated traits such as bone health are avoided. It is clear that more research is needed to quantify the full effect of extending the lay cycle on the birds physiology and welfare. But what is certain is that the industry must be ready to embrace the concept of the long life layer and adapt accordingly.
